# Illinois State Medical Convention

**Published:** 1850-01

**Authors:** 


### ARTICLE IV.

### ILLINOIS STATE MEDICAL CONVENTION.

We heartily approve of the convention of the physicians of this State which is called to meet on the first Tuesday in June next, at Springfield, and hope our professional friends throughout the State will promptly engage in the enterprise and make arrangements to give the meeting a full attendance. It will come at a season of the year when the country is generally healthy, and in this respect the time will be opportune, unless we should again be visited with the cholera. The roads are generally good and the weather pleasant at that season, so that few obstacles will be in the way, and we may hope to see a large delegation of physicians from all parts of the State.

Mutual acquaintance, the promotion of harmony and concert of action, fostering friendly feeling and good fellowship, mutual improvement by interchange of sentiment, and organization for the promotion of the common interests of the profession, may be objects of little importance to some, but we are sure that a large portion of the intelligent members of the profession in Illinois love their calling and its high and benevolent aims too well to lightly regard or neglect them. Besides it is the opinion of the profession of the United States as expressed through the National Medical Association, that such organizations should be formed. E.

